# Mandibular gnathobases of marine planktonic copepods – feeding tools with complex micro- and nanoscale composite architectures

**DOI:** 10.3762/bjnano.6.68

**Published:** 2015-03-06

**Authors:** Jan Michels, Stanislav N Gorb

**Affiliations:** 1 Department of Functional Morphology and Biomechanics, Institute of Zoology, Christian-Albrechts-Universität zu Kiel, Am Botanischen Garten 1–9, D-24118 Kiel, Germany; 2 Biological Oceanography, GEOMAR Helmholtz Centre for Ocean Research Kiel, Düsternbrooker Weg 20, D-24105 Kiel, Germany

**Keywords:** crystalline silica, diatom frustule, mandibular gnathobase, marine planktonic copepod, resilin

## Abstract

Copepods are dominant members of the marine zooplankton. Their diets often comprise large proportions of diatom taxa whose silicified frustules are mechanically stable and offer protection against grazers. Despite of this protection, many copepod species are able to efficiently break even the most stable frustule types. This ability requires specific feeding tools with mechanically adapted architectures, compositions and properties. When ingesting food, the copepods use the gnathobases of their mandibles to grab and, if necessary, crush and mince the food items. The morphology of these gnathobases is related to the diets of the copepods. Gnathobases of copepod species that mainly feed on phytoplankton feature compact and stable tooth-like structures, so-called teeth. In several copepod species these gnathobase teeth have been found to contain silica. Recent studies revealed that the siliceous teeth are complex microscale composites with silica-containing cap-like structures located on chitinous exoskeleton sockets that are connected with rubber-like bearings formed by structures with high proportions of the soft and elastic protein resilin. In addition, the silica-containing cap-like structures exhibit a nanoscale composite architecture. They contain some amorphous silica and large proportions of the crystalline silica type α-cristobalite and are pervaded by a fine chitinous fibre network that very likely serves as a scaffold during the silicification process. All these intricate composite structures are assumed to be the result of a coevolution between the copepod gnathobases and diatom frustules in an evolutionary arms race. The composites very likely increase both the performance of the siliceous teeth and their resistance to mechanical damage, and it is conceivable that their development has favoured the copepods’ dominance of the marine zooplankton observed today.

## Review

### Significance of copepods in marine pelagic food webs

**Figure 1:**
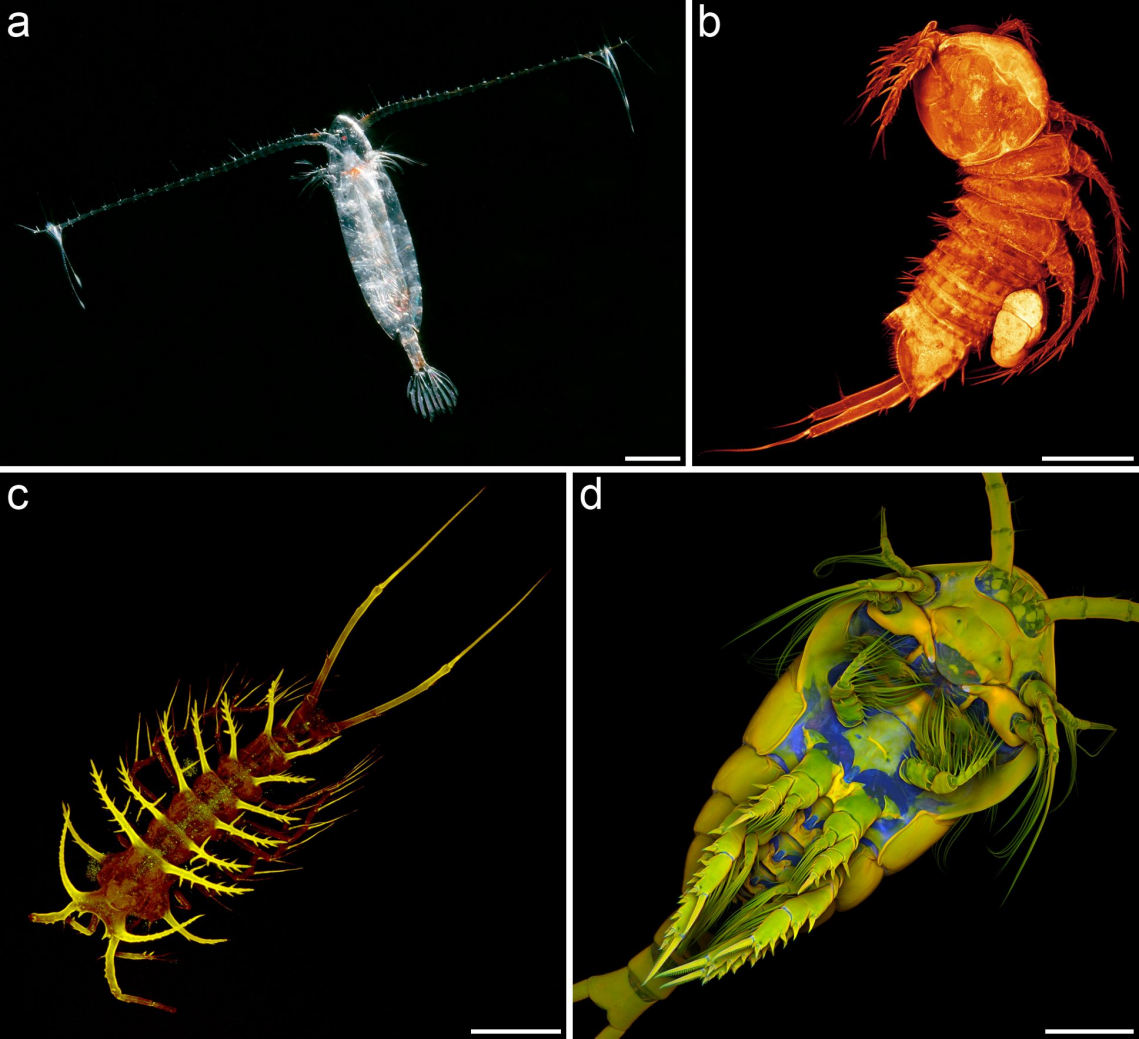
Exemplary copepod species. (a) Female of *Calanoides acutus*, one of the dominant calanoid copepod species within the zooplankton of the Southern Ocean (dorsal view). (b) Female of the harpacticoid copepod genus *Mesocletodes*, collected from deep-sea sediment in the Southern Ocean (lateral view). (c) Female of *Ceratonotus steiningeri*, a harpacticoid deep-sea copepod species, collected from sediment in the Angola Basin at a water depth of 5389 m (dorsal view). (d) Female of the planktonic calanoid copepod species *Temora longicornis*, collected in the North Sea (ventral view). Scale bars = 1 mm (a), 100 µm (b, c), 200 µm (d). (a) Photograph (courtesy of Ingo Arndt). (b–d) Confocal laser scanning micrographs (maximum intensity projections). (b–d) Adapted with permissions from [9–11]. Copyright 2014 Wiley-VCH Verlag GmbH & Co. KGaA.

Crustaceans of the subclass Copepoda (Figure 1) inhabit an impressively large variety of aquatic habitats [1]. In all regions of the earth they can be found in almost any body of water including habitats with extreme conditions such as the deep sea, active hot hydrothermal vents and very cold brine channel systems of sea ice. Copepods are assumed to contribute the largest amount of individuals to the metazoans, even larger than those contributed by insects and nematodes [2–3]. In the marine pelagial the abundance of copepods is particularly pronounced. As a result of this, in all ocean areas worldwide copepods represent the most numerous zooplankton group contributing 55 to 95% of the total zooplankton individuals [4]. The diet of many copepod species contains large proportions of phytoplankton, and copepods are an important food source for various fish species and a large number of other organisms feeding on zooplankton. Accordingly, due to their dominance within the zooplankton, copepods are the main primary consumers and significant links between the primary producers and organisms of higher trophic levels. As such, they represent important food web components and therefore key organisms for processes such as carbon cycling and nutrient regeneration in the marine pelagial [5–6]. In many ocean areas, diatoms account for a large proportion of the phytoplankton ([5,7–8] and citations therein). For this reason they often are an important food source for copepods, and the knowledge of feeding interactions between these two groups of organisms is essential for the understanding of processes related to the food web and energy and particle fluxes in the marine pelagial.

### Mandibular gnathobases – specific feeding tools with morphologies adapted to the diets of the copepods

**Figure 2:**
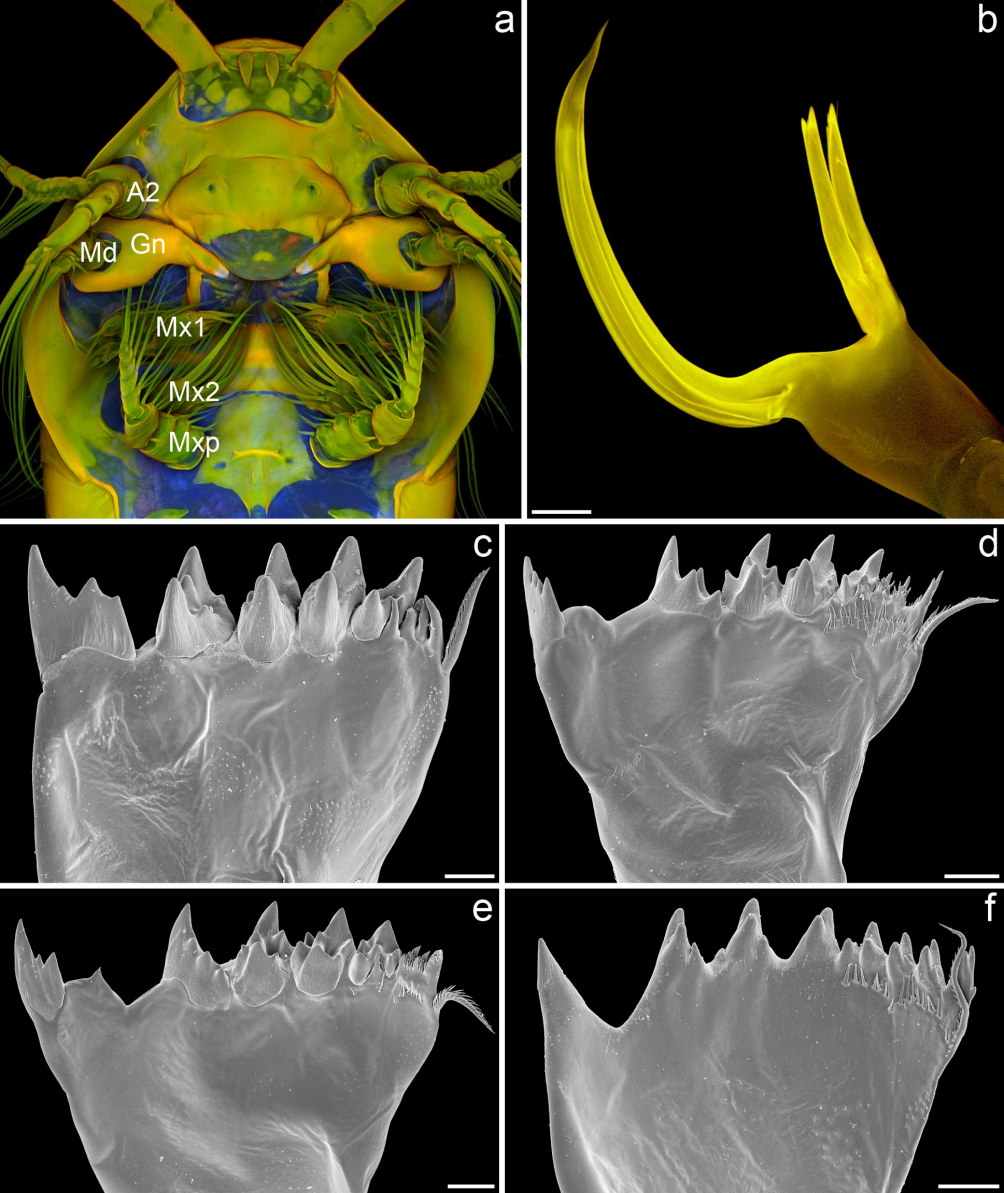
Mouthparts and different types of mandibular gnathobases of calanoid copepods. (a) Section of the micrograph shown in Figure 1d, indicating the location of the five pairs of mouthparts of *Temora longicornis*. A2 = second antenna, Md = mandible, Gn = mandibular gnathobase, Mx1 = first maxilla, Mx2 = second maxilla, Mxp = maxilliped. (Only one mouthpart of each pair is marked.) (b) Confocal laser scanning micrograph (maximum intensity projection) showing the left gnathobase of a male *Heterorhabdus* sp. from the Southern Ocean (cranial view). (c–f) Scanning electron micrographs showing the left gnathobases from females of different Antarctic copepod species (all cranial view). (c) *Rhincalanus gigas*. (d) *Calanoides acutus*. (e) *Calanus propinquus*. (f) *Metridia gerlachei*. Scale bars = 50 µm (b), 25 µm (c–e), 20 µm (f). (b) Adapted with permission from [26].

**Figure 3:**
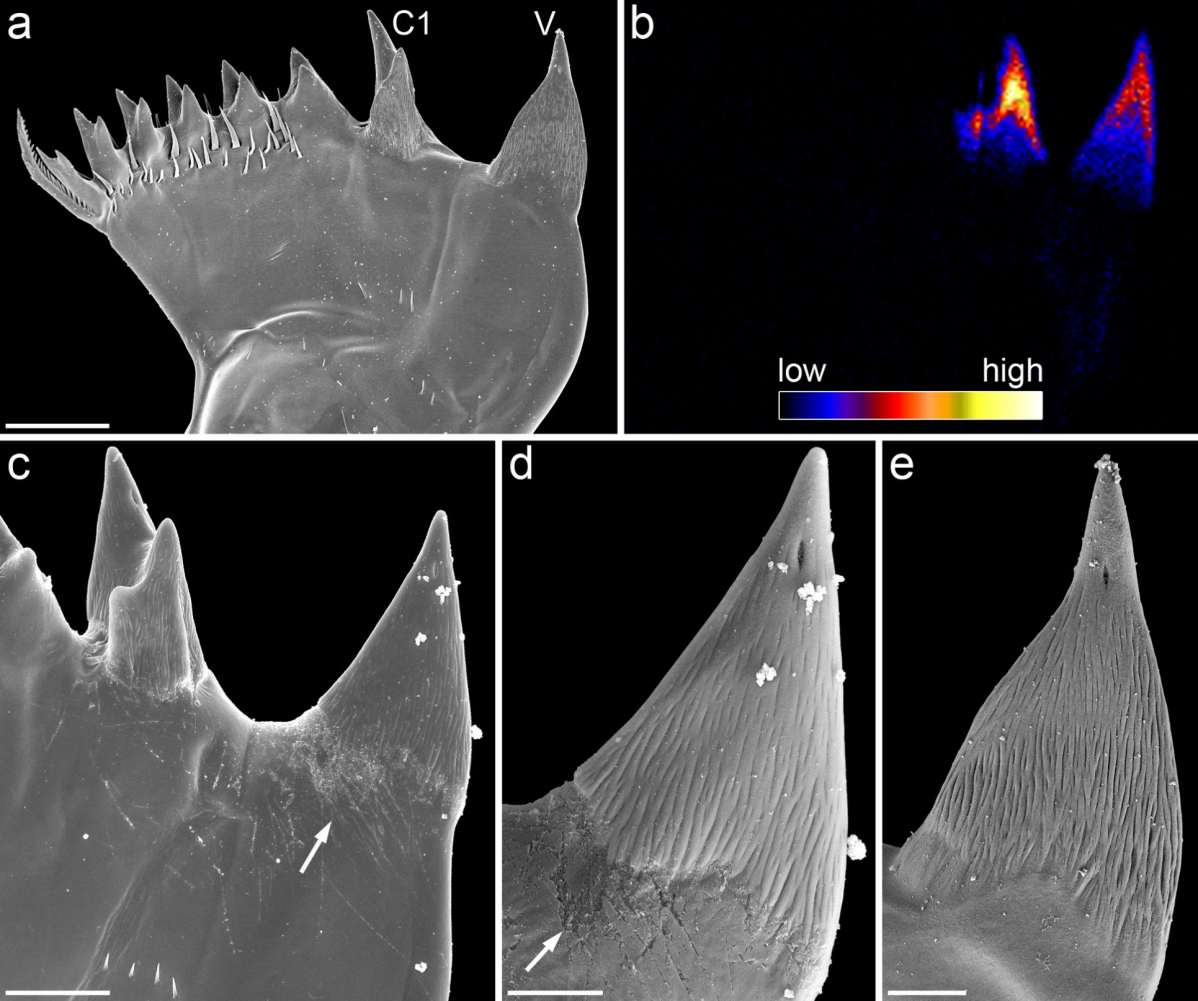
Mandibular gnathobases of female *Centropages hamatus*. (a, c–e) Scanning electron micrographs (all cranial view). (a) Overview of the distal part of a gnathobase. (c) Overview of the ventral part of the distal gnathobase structures. (d) Detailed view of the ventral tooth shown in (c). (e) Detailed view of the ventral tooth shown in (a). (b) Micro-particle-induced X-ray emission (µ-PIXE) mapping showing the distribution and concentration of silicon in the distal part of a gnathobase. The orientation of the gnathobase is similar to that of the gnathobase shown in (a). The results of the elemental analysis indicate that the ventral tooth (V) and the first central tooth (C1) contain silica. The arrows indicate areas with a large number of scratches. Scale bars = 20 µm (a), 10 µm (c), 5 µm (d, e). Figure reproduced with permission from [32].

Copepods usually possess five pairs of mouthparts (Figure 2a), which are used to detect, collect and take up food organisms and particles [12–18]. The mouthparts create water streams, so-called feeding currents, at the ventral side of the copepods’ bodies and scan these currents for food organisms and particles by means of mechanoreceptors and chemoreceptors. After detection, the organisms and particles are evaluated with the aid of these receptors, and the favoured ones are moved to the stoma of the copepods by additional movements of the mouthparts. Subsequently, the food items are grabbed and, if necessary, crushed and minced by the mandibular gnathobases, the basal parts of the mandibles, before being ingested. While, in general, the morphology of the mouthparts differs between species with different diets [19–22], the differences in the morphology of the mandibular gnathobases are particularly pronounced and clearly related to the diet of the respective copepod species [19–21,23–24]. The different gnathobase morphologies can be classified in three main groups: (1) gnathobases of copepods that are carnivorous and feed mainly on other zooplankton organisms have relatively long and sharp tooth-like structures (called ‘teeth’ in the following), and the number of teeth is smaller than those of the gnathobases of the other two groups (Figure 2b); (2) copepod species that mainly feed on phytoplankton possess robust gnathobases with compact and relatively short teeth at their distal ends (Figure 2c–e); (3) omnivorous copepods have gnathobases with a morphology representing an ecotonal form between the morphologies of the other two groups (Figure 2f). The gnathobases of the first group are often rather specialised. Prominent examples for such a specialisation are the gnathobases of the calanoid copepod genus *Heterorhabdus*. They possess only a small number of teeth, and their ventral tooth exhibits a complex morphology that is comparable to the architecture of hypodermic needles and is strongly adapted to catching, anaesthetising and killing prey organisms [25]. This tooth is hollow and features two openings, one at its base and another one at its tip. The lumen of such a tooth is filled with venom or anaesthetic secreted from glandular cells through specific labral pores, which are located close to the opening of the tooth base when the gnathobase is in its ‘inoperative position’ at the labrum. The ventral tooth of the left gnathobase is exceptionally long (Figure 2b), and it is easily conceivable that this tooth can be efficiently used by the carnivorous copepods to spear prey and inject the venom or anaesthetic into its body. In general, the gnathobases of the first group are suitable to pierce and tear apart the prey with their long, pointed and sharp teeth and the reduced number of teeth. By contrast, due to their short, compact and relatively numerous teeth, the gnathobases of the second group seem to be very capable of crushing stable food items such as diatoms. The teeth of these gnathobases have usually been called ‘grinding teeth’ [19]. However, a grinding function of these teeth to crush for example stable diatom frustules is not very conceivable. Many of the respective gnathobase teeth possess small cusps that would clearly decrease the efficiency of such a mechanism. It is much more likely that the copepods crush food items such as diatom frustules by exerting pressure with their gnathobase teeth and thereby concentrating the force on a clearly smaller area by means of the small teeth cusps. Especially in the case of the hollow diatom frustules the application of such a punctual pressure seems to be advantageous over a grinding mechanism and likely leads to a more effective disruption of the frustule structures.

### Mandibular gnathobases with siliceous teeth

In many calanoid copepod species, some of the gnathobase teeth obviously have another material composition than the rest of the gnathobases. This different appearance can easily be shown in an ordinary way by bright-field microscopy, and it becomes clearly evident when the gnathobases are visualized with scanning electron microscopy (e.g., Figures 2c–e, 3a, 3c–e, 5a, 6a). Already several decades ago the application of simple preparation methods and microscopy techniques resulted in the assumption that such teeth are composed of silica [27]. However, it was not until many years later that the presence of gnathobase tooth structures with similar material properties was mentioned and described for additional copepod species [28–30], and not earlier than several additional years later the application of microprobe and electron diffraction analyses confirmed the presence of silica in such teeth [31]. The analyses indicated that the silica is present in the teeth in the form of opal, a hydrated amorphous type of silica. For this reason, the term ‘opal teeth’ was established. The application of both differential interference contrast microscopy and transmission electron microscopy revealed the morphogenesis of the siliceous teeth [31]. They develop early in the pre-moult phase of the moult cycle. After the formation of fibrous tooth moulds, these moulds are connected via ducts to glandular tissue located in the proximal part of the gnathobase. It is assumed that unpolymerised silicic acid is released by this gland tissue and transported inside the ducts to the moulds where the silicification takes place. The final siliceous crown-like or cap-like structures are located on a socket consisting of chitinous exoskeleton material (Figure 6b).

**Figure 4:**
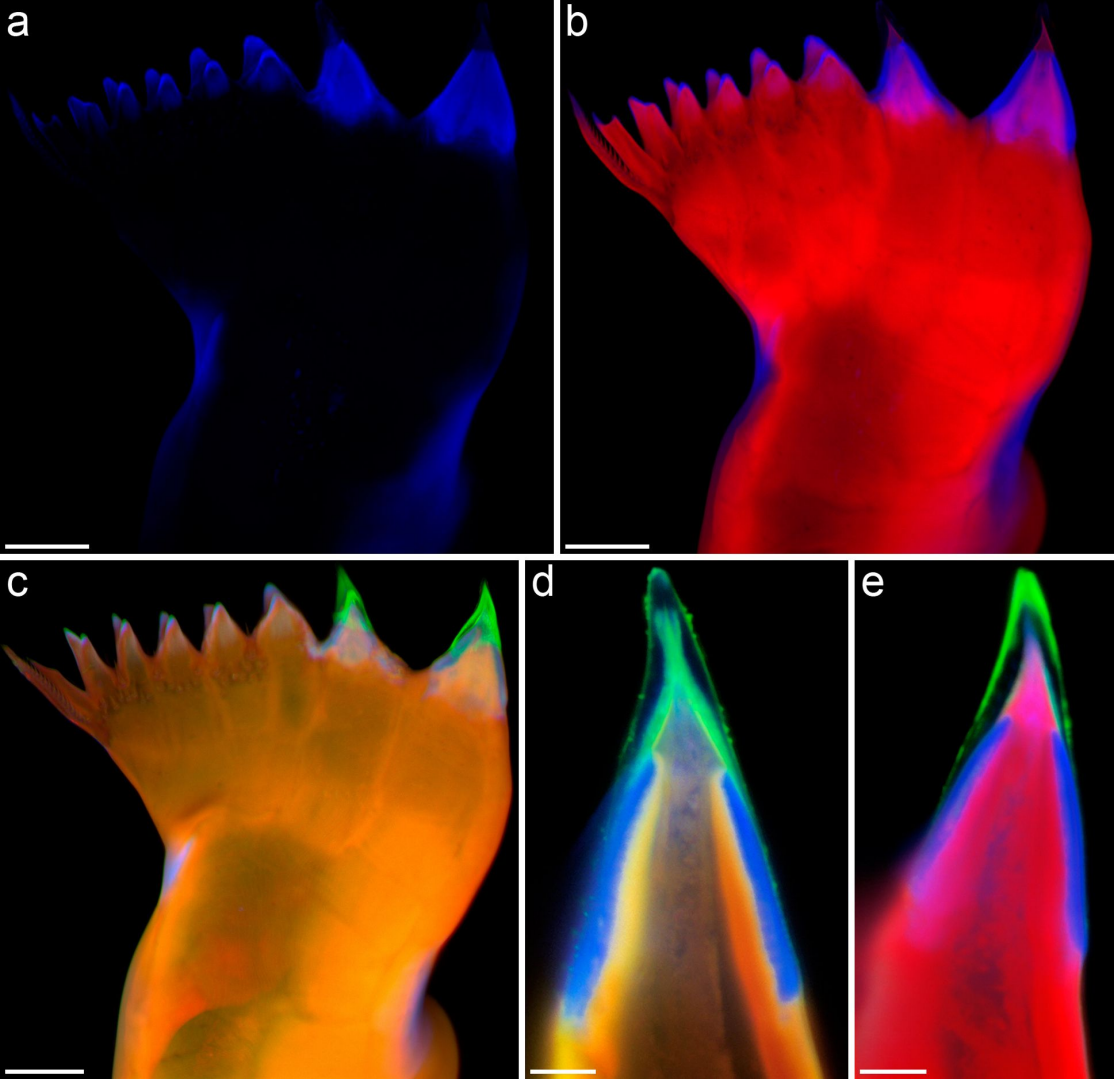
Mandibular gnathobases of female *Centropages hamatus*. (a–e) Confocal laser scanning micrographs (all cranial view) ([a–c] maximum intensity projections showing the whole gnathobase; [d, e] 1-µm-thick optical sections through the ventral tooth). (a) Distribution of resilin. (b) Chitinous exoskeleton (red) and resilin-dominated structures (blue). (c–e) Chitinous exoskeleton (orange, red), resilin-dominated structures (blue, light blue) and silica-containing structures (green). Scale bars = 20 µm (a, b, c), 5 µm (d, e). Figure adapted with permission from [32].

**Figure 5:**
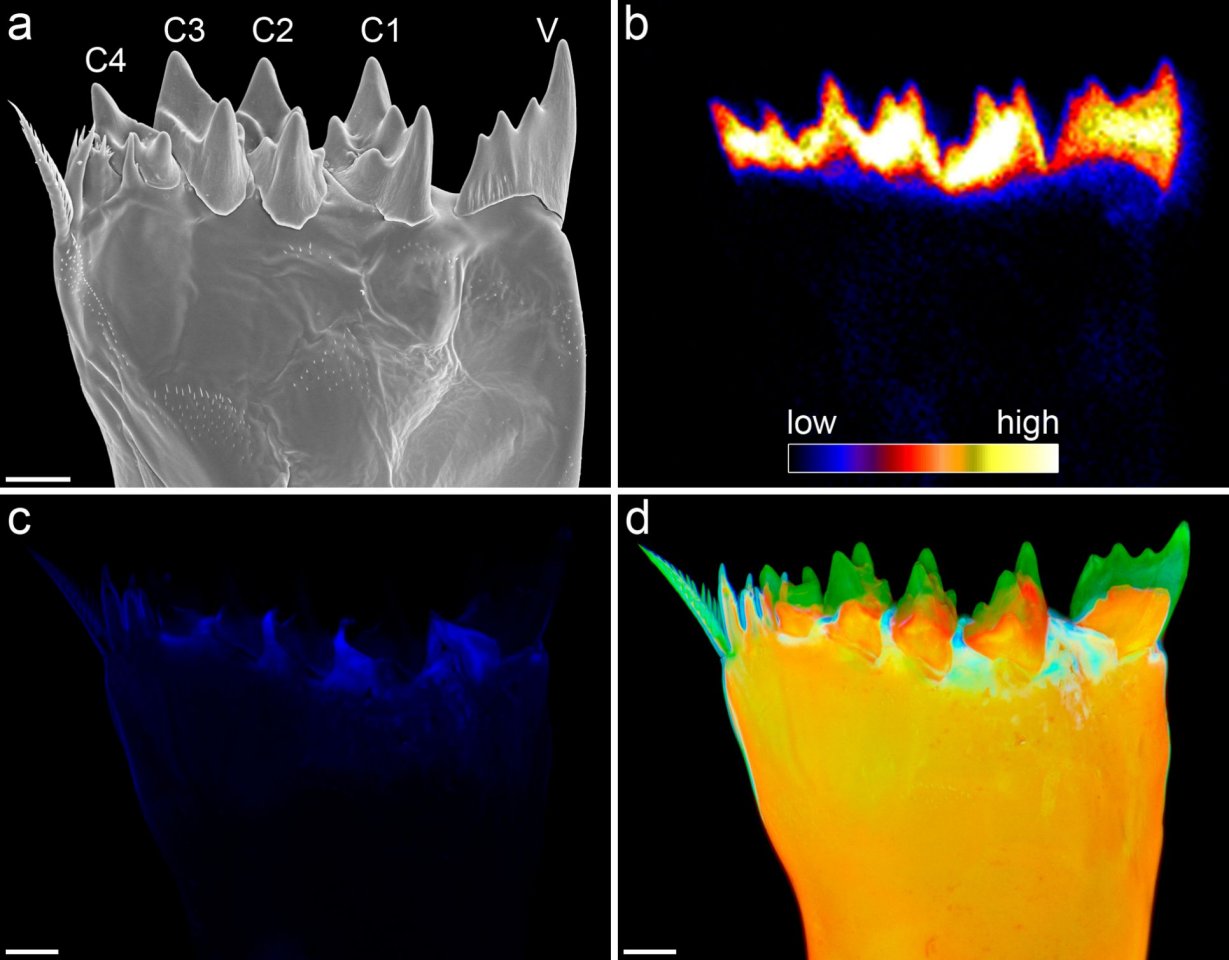
Mandibular gnathobases of female *Rhincalanus gigas*. (a) Scanning electron micrograph showing the distal part of a gnathobase (cranial view). (b) µ-PIXE mapping depicting the distribution and concentration of silicon in the distal part of a gnathobase. The orientation of the gnathobase is similar to that of the gnathobase shown in (a). (c, d) Confocal laser scanning micrographs (maximum intensity projections) showing the material composition of the distal part of a gnathobase (caudal view). (c) Distribution of resilin. (d) Chitinous exoskeleton (orange), resilin-dominated structures (blue, light blue, turquoise) and silica-containing structures (green). The results indicate that the ventral tooth (V) and all central teeth (C1–C4) feature a silica-containing cap-like structure located on top of a chitinous socket. Scale bars = 25 µm. Figure adapted with permission from [33]. Copyright 2015 Elsevier.

Recent studies revealed new insights into the architecture of the siliceous teeth. While the presence of silica in the gnathobase teeth was confirmed with modern high-resolution elemental analysis techniques and confocal laser scanning microscopy [32–34] (Figures 3b, 4c–e, 5b,d), the results of high-resolution transmission electron microscopy analyses clearly indicate that the silica in the gnathobase teeth is composed of only some amorphous silica and large proportions of crystalline silica [33]. Evidence for a crystalline structure of the siliceous teeth had already been mentioned earlier but unfortunately without showing and describing any results [30]. The recent analyses showed that the crystalline silica material present in the siliceous teeth is consistent with the mineral α-cristobalite [33].

**Figure 6:**
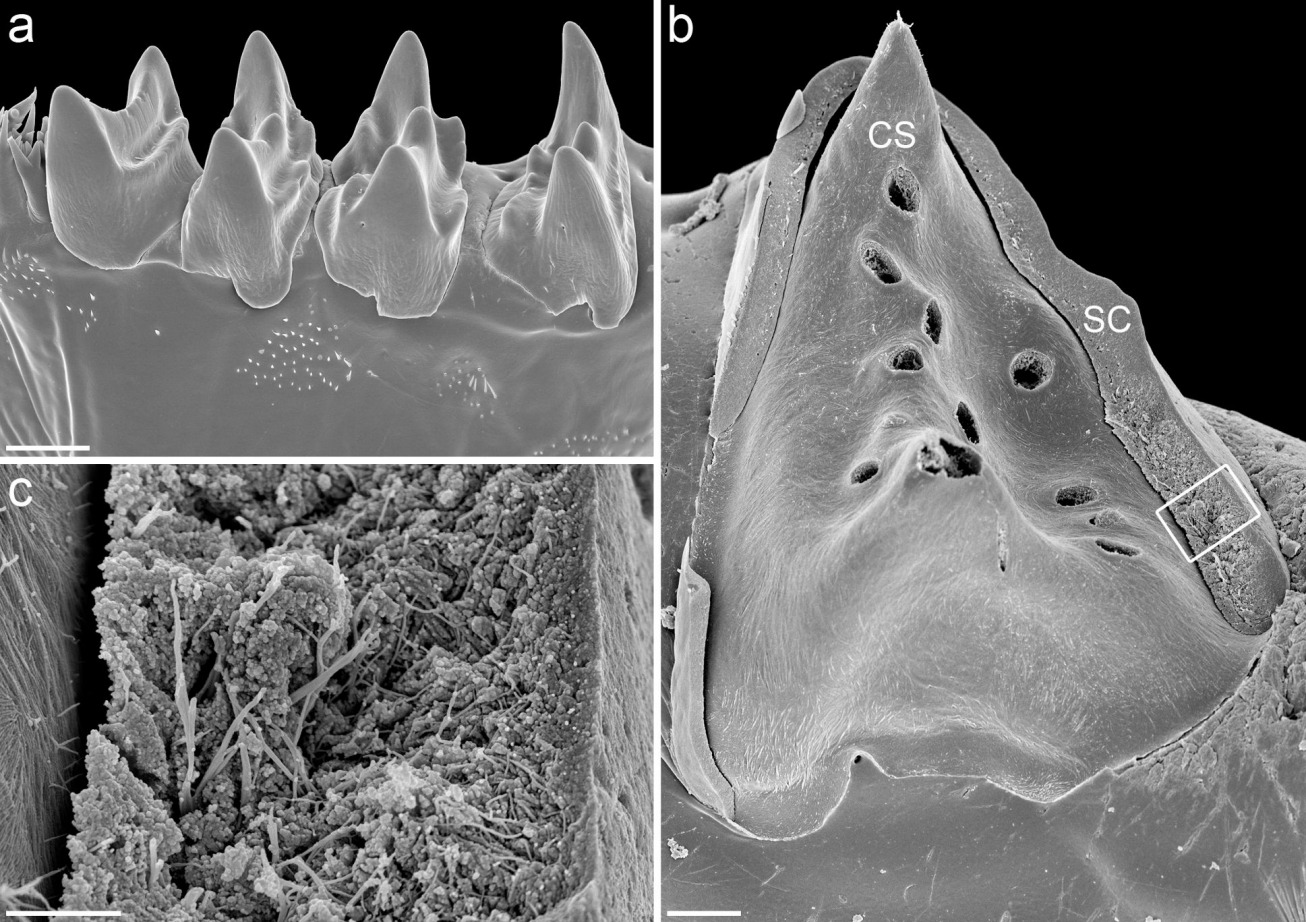
Mandibular gnathobases of female *Rhincalanus gigas*. Scanning electron micrographs (all caudal view). (a) The four central teeth. (b) Central tooth after the removal of large parts of the silica-containing cap-like structure. (c) Detailed view of the structures marked by the rectangular frame in (b). Scale bars = 20 µm (a), 5 µm (b), 1 µm (c). CS = chitinous socket, SC = silica-containing cap-like structure. Figure adapted with permission from [33]. Copyright 2015 Elsevier.

In nature, silica biomineralisation typically takes place on organic matrices composed of compounds such as chitin and collagen that are preferential sites for nucleation and control the formation of the silica structures [35]. Siliceous diatom frustules, for example, contain an internal organic network of cross-linked chitin fibres that is assumed to be a scaffold for silica deposition [36]. After chemical removal of the silica from the gnathobases or fracturing the siliceous cap-like structures, fibre networks become visible in the siliceous gnathobase teeth [33] (Figure 6b,c). The fibres are similar in appearance to those present in the diatom frustules, and they were shown to also be chitinous [33]. It is very likely that the fibre networks serve as templates or scaffolds during the silicification process and are congruent with the fibrous tooth moulds mentioned above.

The silica-containing structures in the copepod gnathobases likely increase the mechanical strength and stability of the gnathobase teeth, and they are assumed to have coevolved with the siliceous diatom frustules [32,37]. For copepod species that mainly feed on phytoplankton this is certainly conceivable. However, the presence of silica in gnathobase teeth of carnivorous copepods [25] suggests that siliceous teeth represent an adaptation to frequent mechanical loads in general. In this context, the degree of silicification seems to be related to the mechanical stability of the main food items and thereby to the intensity of the prevalent loads. In siliceous teeth such as the cannula-like ones of *Heterorhabdus* spp., which are likely exposed to relatively moderate forces only, the silica-containing structures are relatively small and not particularly pronounced [25]. By contrast, siliceous teeth regularly facing strong mechanical interactions with diatom frustules, which can be mechanically very stable [37] and therefore cause high forces affecting the teeth during feeding, typically have very pronounced silica-containing structures, which seem to be rather compact and stable [24] (Figures 2c–e, 5a, 6a). For this reason a coevolution between diatom frustules and gnathobases with very pronounced siliceous teeth is very likely.

The question regarding the origin of the silica in the gnathobase teeth arose already relatively long ago [28]. There are two potential sources. The copepods could either take up silicic acid from the seawater where it is present in all ocean areas [38–39], or they could utilise the silica that they ingest when they feed on diatoms or other copepods with siliceous teeth for the formation of their own siliceous teeth. Laboratory experiments showed that the copepods take up silicic acid from the seawater and are able to cover their silicon demand for the formation of siliceous teeth even at rather low silicic acid concentrations [30]. The results indicate that the lowest natural marine silicic acid concentrations, found in oligotrophic ocean areas, are still high enough to sufficiently supply the copepods with silicon. Nevertheless, it is imaginable that the copepods use both potential sources and, besides taking up silicic acid from the seawater, also extract silicon from their diet where it is often present in high concentrations and therefore represents an efficient source. However, this hypothesis has never been investigated so far. In a respective experiment the frustules of living diatoms could be labeled with the radioisotope ^32^Si, and the diatoms could be fed to copepodids (juvenile copepods) to test if ^32^Si is included in the siliceous teeth of the copepods after moulting.

Up to now the mechanical stability of the silica-containing structures of gnathobase teeth has not been analysed. Such an analysis could potentially be performed using nanoindentation. However, because of the small dimensions of the structures it would be rather difficult to get reliable results. For insect mandibles, many of which are known to contain relatively high concentrations of zinc and manganese [40–41], it has been shown that the metal incorporations increase the hardness of the mandible material [42–43]. Copepod gnathobases often exhibit scratches caused by contact with hard food items. Interestingly, these scratches are typically only found on the surfaces of the chitinous material while the surfaces of the siliceous structures seem to be resistant to such abrasive damage (Figure 3c,d). This indicates that the presence of silica very likely increases the hardness and stiffness of the gnathobase teeth and therefore has a similar effect as zinc and manganese have in insect mandibles.

### Mandibular gnathobases, diatom frustules and the evolutionary arms race

In addition to the presence of mechanically stable silica-containing structures, recent detailed analyses of the material composition of copepod gnathobases yielded further indication of a coevolution between diatom frustules and very pronounced siliceous teeth. The respective analyses had been inspired by the knowledge that structures consisting of hard materials easily break because of local stress concentrations under high mechanical loads when they are in contact with other hard structures [44]. To test the idea that the non-siliceous gnathobase parts might have evolved specific properties that reduce the risk of wear and damage of the siliceous teeth, the materials embedding and bearing these teeth were recently investigated in the two calanoid copepod species *Centropages hamatus* and *Rhincalanus gigas*, both of which have diets with significant proportions of diatoms [32–33]. Interestingly, in the gnathobases of both species exoskeleton structures with high proportions of the elastic protein resilin were discovered. The results show that the architecture and the composition of the composite structures in the gnathobase teeth are much more complex than previously assumed. In *C. hamatus*, the siliceous teeth feature a cap-like structure that contains high resilin proportions. This structure is located on top of a chitinous socket and covered by another cap-like structure containing silica (Figure 4). The siliceous teeth of *R. gigas* are characterised by a silica-containing cap-like structure that is situated on top of a chitinous socket (Figure 5d). At the bases of the sockets of the siliceous teeth, the gnathobase exoskeleton features high proportions of resilin (Figure 5c,d), while, by contrast, in the central and proximal parts of the gnathobase the exoskeleton is dominated by chitinous material.

Compared with chitinous exoskeleton material, resilin is very soft and elastic [45–46]. At first view it might be surprising that hard and stiff structures, which are supposed to be adapted to crushing stable diatom frustules, are combined with very soft structures. When the copepods feed on diatoms, local stress concentrations caused by mechanical loads on the tips of the siliceous teeth might exceed the breaking stress level and thereby increase the risk of crack formation in and breakage of the teeth. In comparable situations, when mechanical systems have to resist severe mechanical challenges, a subtle combination of materials with different mechanical properties (or a gradient in the material properties) can make these systems more resistant to damage and wear because such an architecture minimises the probability of local stress concentrations and, in the case of an initial damage, prevents further crack propagation [47–48]. It is conceivable that the soft and elastic resilin-dominated structures of the siliceous teeth function as flexible supports of the hard and stiff tooth structures. In case the breaking stress level is reached, these structures might be deformed by compression and thereby reduce stress concentrations in the tooth material. Such a mechanism likely improves the resistance of the siliceous teeth to mechanical damage. In *C. hamatus*, additional structures with high resilin proportions, located at the dorsal edge of the central part and at the ventral edge of the proximal part of the gnathobases (Figure 4a–c), might function as a cushioning system that makes the whole gnathobases resilient and thereby further reduces the risk that the siliceous teeth are mechanically damaged.

Diatoms, which often feature complex frustule architectures that very likely have evolved to increase the mechanical stability of the frustules and provide resistance to compression loads applied to the frustules from outside [37], represent the most stable food items found in copepod diets. Intact diatoms can survive the passage through the guts of zooplankton organisms [49]. For this reason being able to crush and mince the diatom frustules is important for the copepods to better digest the diatom cells. However, successful crushing and mincing of such mechanically protected frustules requires specifically adapted feeding tools. Accordingly, the presence of very complex composite tooth structures containing diverse materials such as resilin and silica supports the assumption that the respective siliceous copepod teeth have specifically coevolved with the stable diatom frustules in an evolutionary arms race (for the explanation of the term ‘arms race’ see [50]) and enable the copepods to more efficiently feed on and utilise their main food organisms.

**Figure 7:**
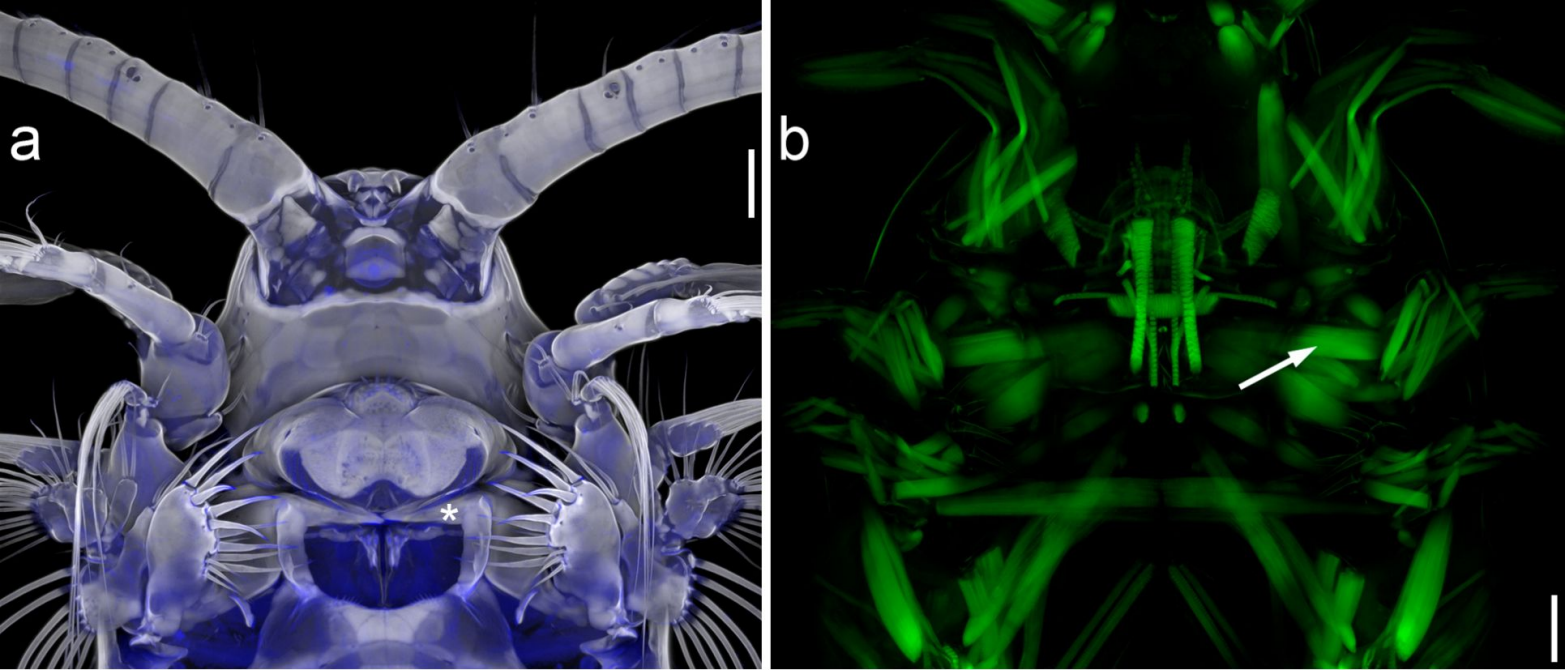
Muscular system of the anterior part of *Centropages hamatus*. Confocal laser scanning micrographs (maximum intensity projections) showing ventral views of the exoskeleton (a) and the muscles (b) of female *C. hamatus*. Please note that the two micrographs show different sections of two different copepod specimens. The asterisk and the arrow indicate the positions of the left gnathobase and the strong muscles of the left mandible, respectively. Scale bars = 50 µm. Figure reproduced with permission from [10]. Copyright 2014 Wiley-VCH Verlag GmbH & Co KGaA.

**Figure 8:**
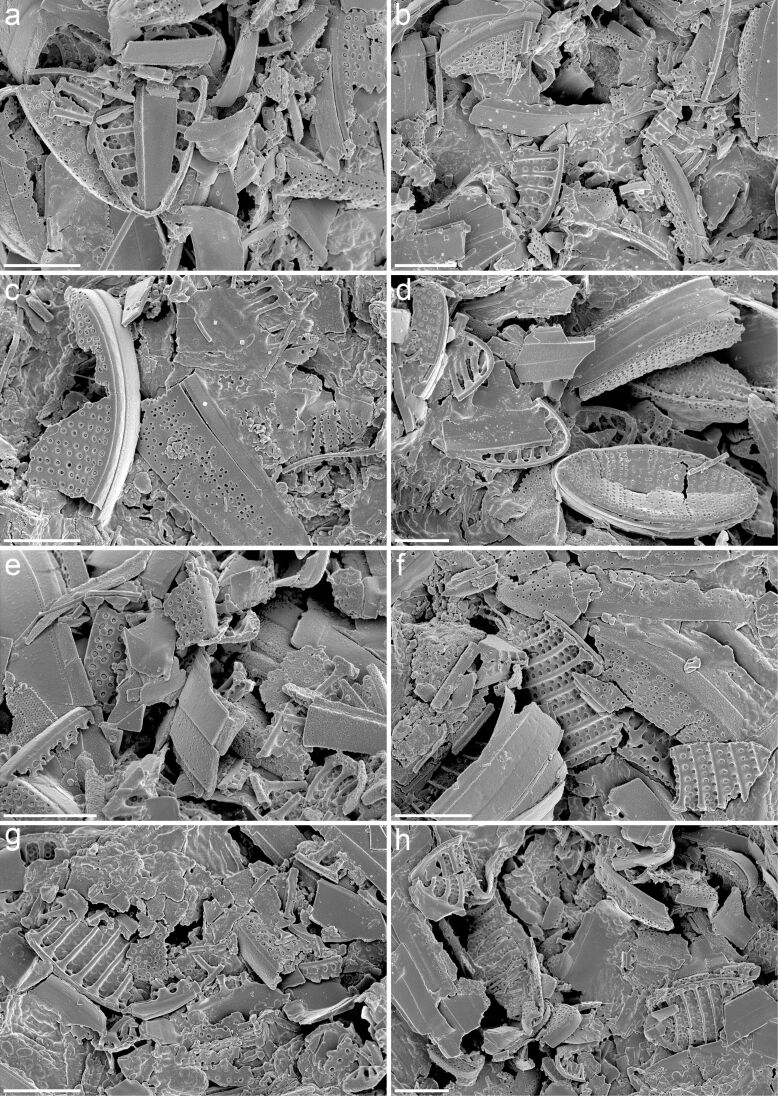
Faecal pellets from feeding experiments with the diatom species *Fragilariopsis kerguelensis* and juveniles (copepodite stage V) of the Antarctic copepod species *Calanus propinquus*. (a–h) Scanning electron micrographs showing pieces of *F. kerguelensis* frustules present in the faecal pellets. Scale bars = 5 µm.

Protection against specific ‘attack systems’ of grazers and predators is assumed to be the main factor that controls plankton evolution having resulted in the existence of a large variety of morphologies and chemical and mechanical defence systems [51]. In this context, the copepod gnathobases featuring hard and stable biomineralised tooth structures with soft and elastic supports represent examples of highly-adapted ‘attack systems’. A powerful operation of the gnathobases is likely ensured by pronounced mandibular muscles (Figure 7). This combination might be a prerequisite for the copepods’ documented ability to crush and mince the diatom frustules into small pieces [52–53]. Large copepods such as the Antarctic species *Calanus propinquus* with pronounced siliceous teeth (Figure 2e) are capable of destroying even the frustules of the diatom *Fragilariopsis kerguelensis* (Figure 8) that are particularly stable [37]. The copepods’ effective crushing and mincing of diatom frustules certainly not only depend on the morphology and the material composition of the gnathobases but are also related to the dimensions of the copepods and their diatom food. In feeding experiments, for example, the relatively small copepod *Acartia clausi* was observed to damage only frustules of the small size fraction of the diatoms offered, while the larger species *Centropages hamatus* and *Temora longicornis* were able to also damage the frustules of the large size fractions [53].

Besides their morphological adaptations, copepods exhibit specific feeding techniques and strategies enabling them to better utilise the available diatom food. Frustules of large diatoms such as *Coscinodiscus wailesii* are not always completely destroyed and ingested during feeding. *T. longicornis* was observed to break only small pieces out of the *C. wailesii* frustules, and subsequently it ingested the cell contents and dropped the frustules [54]. In other experiments, *C. hamatus* exhibited a similar feeding strategy. While smaller *C. wailesii* frustules were broken in pieces, the large frustules were only ‘opened’ by breaking a hole in the girdle band, which was shown to be the frustules’ weakest part [53].

In general, the adapted gnathobase morphologies and material compositions combined with effective feeding techniques and strategies make copepods very powerful antagonists of diatoms in the evolutionary arms race. Copepod features such as the shape of the body, the antennae equipped with a high amount of sensors, powerful muscles enabling exceptional escape jumps, the capability to remotely detect and capture prey and efficient mate finding are assumed to be the basis for the success of the marine planktonic copepods [55]. Nevertheless, it is conceivable that the development of the complex composite gnathobase structures that are adapted to efficiently capturing (or grabbing), crushing and mincing food items also accounts considerably for the dominance of the copepods observed today within the marine zooplankton.
